# Early-life conditioning strategies to reduce dietary phosphorus in broilers: underlying mechanisms

**DOI:** 10.1017/jns.2020.17

**Published:** 2020-07-06

**Authors:** A. S. Valable, M. P. Létourneau-Montminy, S. Klein, L. Lardic, F. Lecompte, S. Metayer-Coustard, N. Même, G. Page, M. J. Duclos, A. Narcy

**Affiliations:** 1 Animal Sciences Department, Université Laval, 2425 rue de l'Agriculture, QC, Canada G1V 0A6; 2 INRAE, Université de Tours, UMR BOA, 37380 Nouzilly, France; 3 MiXscience, Centre d'affaires Odyssée, ZAC Cicé Blossac, 35172 Bruz, France; 4 Plateforme CIRE, service imagerie – UMR 0085 PRC, INRA centre val de Loire, 37380 Nouzilly, France; 5 Trouw Nutrition Agresearch, Ontario, Canada

**Keywords:** Broiler chickens, Feeding strategies, Calcium, Phosphorus, Adaptation, AID, apparent ileal digestibility, BW, body weight, H, high non-phytate P and Ca, HU, Hounsfield units, L, low non-phytate P and Ca, nPP, non-phytate P, PPdisp, disappearance of phytate P

## Abstract

Chickens adapt to P and Ca restriction during the very first days of life by improving P utilisation efficiency. The present study was built to identify the mechanisms underlying this adaptive capacity, and to identify the optimal window of application of the restriction (depletion). A total of 1600 Cobb 500^TM^ male broilers were used. During each phase (from age 0 to 4 d, 5 to 8 d, 9 to 18 d and 19 to 33 d), the animals received either a control diet (H) or a restricted diet (L) with reduced levels of non-phytate P (nPP) and Ca (between −14 and −25 % for both) with four dietary sequences: HHHH, HLHL, LHHL and LLHL. None of the feeding strategies affected growth. Tibia ash content at day 4 and 8 was impaired when the L diet was fed from 0 to 4 and 5 to 8 d, respectively (*P* = 0⋅038 and *P* = 0⋅005). Whatever the early restriction period or length between 0 and 8 d of age, the mineralisation delay was compensated by day 18. This was accompanied by an increased mRNA expression of the Ca transporter, *CALB1*, and an increased apparent ileal digestibility of Ca at day 8 (*P* < 0⋅001). This adaptation was limited to the starter phase in restricted birds. No effect was seen on P transporters mRNA or protein expression. In conclusion, birds adapted to mineral restriction by increasing Ca and nPP utilisation efficiencies. Depletion−repletion strategies are promising in improving the sustainability of broiler production but need to be validated in phytase-supplemented diets.

Nutritional strategies for improving the efficiency of P use by birds could be implemented to reduce P excretion and avoid P overfeeding. The reduction of dietary P level is possible if the Ca level is simultaneously decreased^(1)^ due to lower propensity for Ca–phosphate and Ca–phytate complexes in the digestive tract. This strategy is particularly interesting in the finisher phase where feed intake and consequently P excretion are high. In addition, broilers fed diets deficient in Ca and non-phytate P (nPP) during an early phase of growth (depletion) exhibit better P and Ca utilisation and are able to compensate for bone mineralisation^(1,2)^. A more complete understanding of the mechanisms involved in this adaptation is required to efficiently use this kind of strategy.

Intestinal enterocytes are constantly exposed to fluctuations in Ca and/or P availability. These fluctuations have been proven to affect gene and protein expression and the activity of transporters through a mechanism driven by parathormone and vitamin D_3_^(3)^. At low nPP and Ca intake, birds have been shown to adapt by increasing mRNA expression of Ca transporters: *CALB1*^(4)^, *ATP2B1*^(5,6)^ and *SLC8A1*^(6)^ and P transporters: *SLC34A2*^(7,8)^ and *SLC20A1*^(9)^ in the small intestine. So far, the majority of these studies have focused on the mRNA expression of no more than three genes at a time, focusing mainly on either Ca or P transporters. Moreover, to our knowledge, only a few studies have measured the impact of these diets on the protein expression of P^(9,10)^ and Ca^(4,6)^ transporters in broilers.

Ashwell & Angel^(11)^ observed that birds fed a deficient diet during the first 90 h of life improved P and Ca apparent absorption compared with control birds. This adaptation was apparent from 90 h and remained even if the birds were fed an adequate diet from 90 h to 38 d of age. It was linked to an increase in *SLC34A2* mRNA expression. Thus, those authors made the hypothesis that an imprinting of the birds was possible, involving a long-term adaptation in gene expression. A recent study (MM Van Krimpen, E Willems and HJ Van Harn, unpublished results) failed to observe such a long-term adaptation in birds fed a deficient Ca and nPP diet from 0 to 4 d of age, which showed decreased P and Ca pre-caecal digestibility at day 37, without any variation in the mRNA expression of *CALB1*, *ATP2B1* and *SLC34A2*. These conflicting results suggest a lack of knowledge about the degree of dietary nPP and Ca reduction, the timing and/or the duration of the depletion required to induce a long-term adaptation in the birds.

The first objective of this experiment was to identify the optimal depletion−repletion strategies. Several conditions (duration and timing) were tested during the depletion phase with the hypothesis that the birds could undergo a long-lasting adaptation with an improved efficiency in mineral utilisation while maintaining growth and bone mineral status. The second objective was to identify and understand the mechanisms related to the adaptation with the hypothesis that it could involve modifications in Ca and P duodenal transporter mRNA and/or protein expression.

## Materials and methods

The experiment was conducted under the guidelines of the French Ministry of Agriculture for Animals (Paris, France, authorisation 1153-2015071514404178) at PEAT INRA Poultry Experimental Facility (2018, https://doi.org/10.15454/1.5572326250887292E12).

### Experimental diets

The experimental period was divided into four phases: from 0 to 4, 5 to 8, 9 to 18, and 19 to 33 d of age. All experimental diets were based on maize, soyabean meal and wheat. During each phase, the birds received either a high (H) or a low (L) diet (Table 1). The Ca and nPP levels in the H diets were in accordance with the breeder recommendations^(12)^ between 0 and 4 d of age and were at the levels used in commercial conditions for the other phases. The L diets were designed to contain between 14 and 25 % lower Ca and nPP levels compared with the H diet, depending on the phase. For the first three phases, the Ca content was, respectively, 1⋅00, 0⋅92 and 0⋅77 % in the H diets and 0⋅77, 0⋅70 and 0⋅66 % in the L diets. Likewise, nPP contents were, respectively, 0⋅45, 0⋅45 and 0⋅35 % in the H diets and 0⋅35, 0⋅32 and 0⋅30 % in the L diets. The last phase was divided into two dietary periods: 19 to 28 d of age and 29 to 33 d of age. The Ca and nPP contents in the H diet were, respectively, 0⋅64 and 0⋅29 % from 19 to 28 d of age and then 0⋅55 and 0⋅25 % from 29 to 33 d of age. The Ca and nPP contents in the L diet were, respectively, 0⋅53 and 0⋅24 % from 19 to 28 d of age and then 0⋅44 and 0⋅20 % from 29 to 33 d of age. Diets in each phase were combined to form four dietary sequences: (1) HHHH; (2) HLHL; (3) LHHL; and (4) LLHL (see Table 2). The first treatment was the control. Treatments 2, 3 and 4 were designed to study the timing and duration of the first depletion (from 0 to 8 or 0 to 4 or 5 to 8 d of age). During the finisher phase, feed intake and P excretion are at their highest levels. Therefore, the P and Ca levels were reduced during this period based on a previous study indicating the possibility of a complete recovery of the birds at the bone level^(1)^. Calcium carbonate (CaCO_3_) and dicalcium phosphate (CaHPO_4_) were added to the diets to adjust the Ca and nPP content. Titanium dioxide (TiO_2_; 0⋅3 %) was added to the diets from 5 to 18 d of age and from 28 to 33 d of age as an indigestible marker to enable determination of P and Ca apparent ileal digestibility (AID). Diets and water were distributed *ad libitum*. Diets were distributed as medium crumbs from 0 to 4 d of age and as medium pellets after day 5.

**Table 1.** Composition and chemical analysis of the experimental diets (as-fed basis; g/kg unless otherwise stated)

Dietary treatments…	0 to 4 d of age		5 to 8 d of age		9 to 18 d of age		19 to 28 d of age		29 to 33 d of age	
	H	L	H	L	H	L	H	L	H	L
Ingredients										
Maize	428⋅0	442⋅0	387⋅9	402⋅3	436⋅3	443⋅5	470⋅0	477⋅1	494⋅9	502⋅0
Soyabean meal	368⋅0	366⋅0	400⋅1	398⋅0	358⋅2	357⋅1	330⋅5	329⋅5	303⋅4	302⋅3
Wheat	150⋅0	150⋅0	150⋅0	150⋅0	150⋅0	150⋅0	150⋅0	150⋅0	150⋅0	150⋅0
Soya oil	16⋅00	11⋅50	24⋅60	19⋅90	22⋅60	20⋅30	23⋅80	21⋅33	25⋅60	23⋅30
Dicalcium phosphate	17⋅80	12⋅20	15⋅50	9⋅94	12⋅10	9⋅28	9⋅00	6⋅24	7⋅10	4⋅32
Calcium carbonate	3⋅70	1⋅70	6⋅73	4⋅68	5⋅40	4⋅44	4⋅25	3⋅35	3⋅50	2⋅54
Cellulose	4⋅00	4⋅00	5⋅00	5⋅00	5⋅00	5⋅00	5⋅00	5⋅00	5⋅00	5⋅00
Vitamin and mineral premix*	3⋅10	3⋅10	2⋅77	2⋅75	2⋅67	2⋅66	2⋅62	2⋅61	2⋅56	2⋅56
Sodium chloride	2⋅60	2⋅60	2⋅42	2⋅40	2⋅23	2⋅21	2⋅06	2⋅06	1⋅91	1⋅90
dl-Met	0⋅70	0⋅80	0⋅75	0⋅78	1⋅05	1⋅06	1⋅19	1⋅21	1⋅33	1⋅36
Lys HCl	0⋅50	0⋅50	0⋅20	0⋅19	0⋅23	0⋅23	0⋅28	0⋅28	0⋅31	0⋅32
l-Threonine	0⋅60	0⋅60	0⋅83	0⋅86	1⋅02	1⋅02	1⋅10	1⋅12	1⋅19	1⋅20
Anticoccidial	5⋅00	5⋅00	0⋅20	0⋅20	0⋅20	0⋅20	0⋅20	0⋅20	0⋅20	0⋅20
Titanium dioxide (0⋅05%)	0⋅00	0⋅00	3⋅00	3⋅00	3⋅00	3⋅00	0⋅00	0⋅00	3⋅00	3⋅00
Calculated (analysed) content†										
Metabolisable energy (MJ/kg)	12⋅1	12⋅2	12⋅1	12⋅1	12⋅4	12⋅4	12⋅6	12⋅6	12⋅8	12⋅8
Crude protein (%)	21⋅5 (22⋅1)	21⋅5 (22⋅0)	21⋅5 (21⋅2)	21⋅5 (22⋅5)	20⋅0 (20⋅8)	20⋅0 (21⋅2)	19⋅0 (19⋅8)	19⋅0 (19⋅9)	18⋅0 (18⋅6)	18⋅0 (18⋅7)
Ca	10⋅0 (9⋅1)	7⋅7 (7⋅9)	9⋅2 (10⋅1)	7⋅0 (7⋅4)	7⋅7 (7⋅2)	6⋅6 (6⋅3)	6⋅4 (6⋅3)	5⋅3 (5⋅3)	5⋅5 (5⋅2)	4⋅4 (4⋅1)
Total PP	7⋅0 (6⋅5)	6⋅0 (5⋅9)	6⋅7 (6⋅7)	5⋅7 (5⋅6)	5⋅9 (5⋅8)	5⋅4 (5⋅3)	5⋅3 (5⋅4)	4⋅8 (4⋅8)	4⋅8 (4⋅5)	4⋅3 (3⋅9)
nP	4⋅6	3⋅6	4⋅2	3⋅2	3⋅5	3⋅0	2⋅9	2⋅4	2⋅5	2⋅0
Ca:nPP	2⋅2	2⋅1	2⋅2	2⋅2	2⋅2	2⋅2	2⋅2	2⋅2	2⋅2	2⋅2

H, high P and Ca; L, low P and Ca; nPP, non-phytate P.

* Premix, provided per kg of diet: 15 000⋅00 IU vitamin A; 4300⋅00 IU cholecalciferol; 100⋅00 IU dl-α-tocopherol; 5⋅00 mg menadione; 5⋅00 mg thiamine; 8⋅00 mg riboflavin; 100⋅00 mg niacin; 25⋅00 mg calcium pantothenate; 7⋅00 mg pyridoxine; 0⋅30 μg biotin; 3⋅00 mg folic acid; 0⋅03 μg cyanocobalamin; 50⋅00 mg Fe; 20⋅00 mg Cu; 80⋅00 mg Mn; 90⋅00 mg Zn; 2⋅00 mg iodine, 0⋅20 mg Se, 550⋅00 mg choline chloride.

† Calculated from the National Research Council^(34)^. Analysed values in parentheses.

**Table 2.** Experimental design

Treatment name	Diets received per phase			
	0 to 4 d	5 to 8 d	9 to 18 d	19 to 33 d
HHHH	H	H	H	H
HLHL	H	L	H	L
LHHL	L	H	H	L
LLHL	L	L	H	L

H, high non-phytate P and Ca; L, low non-phytate P and Ca.

### Birds and management

A total of 1600 1-d-old male broilers (Cobb 500^TM^) were raised from 0 to 35 d of age. At their arrival, broilers were weighed and randomly allocated to thirty-two pens (eight pens per treatment). The study consisted of a randomised block design with eight blocks of four pens each (3 m^2^ per pen). In each block, pens were randomly allocated to a treatment (400 birds per treatment). The room temperature was settled at 33°C upon arrival and decreased progressively to reach 20°C at day 35. Birds were kept under 24 h light on day 1, 23 h from day 2 to day 4 and 18 h thereafter. The broilers were weighed individually at days 0, 8, 18 and 33 (mean weight 2356⋅1 (sd 230⋅2) g). Feed intake per pen was recorded for each phase. Pre-caecal digesta samples from the distal half of the ileum (defined as extending from Meckel's diverticulum to the ileo-caecal junction) were collected from eight birds per pen (eight pens per experimental diet) at days 8, 18 and 33 after pentobarbital injection. Digesta samples were pooled in order to achieve eight pools of eight birds for each treatment. Out of those sixty-four broilers per treatment, twelve broilers per treatment were used to collect intestinal tissue samples immediately after death. The duodenum was isolated and thoroughly washed in ice-cold PBS solution. A 5-cm medial sample was snap-frozen and scraped into liquid N_2_ before storage at −80°C. The right tibia was collected from twelve birds per treatment at day 4, day 8, day 18 and day 33. At day 33, ten additional birds per treatment were euthanised and stored at −20°C until scanning. The litter from four pens per treatment was weighed at the beginning and the end of the experiment. On the last day of the experiment, a 1⋅5 kg sample was taken after mixing the whole litter to estimate P excretion.

### Computed tomography scans

The carcasses at day 33 were scanned using a clinical computed tomography machine (Somatom Definition AS128; Siemens) with scan parameters set at 140 kV tube voltage and 500 mAs current. The image acquisition mode was a matrix size of 32 cm and 512 px and a resolution of 650 μm. The images were converted into DICOM (Digital Imaging and Communications in Medicine) format for analysis using SINGO.VIA (Siemens) software. Bone mineral density was estimated on the right tibia. The bone mineral density was given in Hounsfield units (HU) by the scanner software and later converted to g/cm^3^ using a phantom (Electron Density Phantom (Model 062 M); Meditest). Total bone was defined in the intervals 200−3000 HU according to Militist *et al*.^(13)^. Trabecular and cortical bones were defined in the intervals, respectively, of 200−800 HU and 801−3000 HU, according to Sherlock *et al*.^(14)^.

### Chemical and mechanical analysis

Litter and ileal digesta samples were freeze-dried. DM was determined for right tibias (103°C for 12 h) and diets (103°C for 4 h). Samples of the diets, ileal contents, litters and tibias were ashed at 550°C for 12 h. For mineral determination, 0⋅5 g of ashes were solubilised in 6 ml HNO_3_ and 4 ml of H_2_SO_4_ and treated by microwave (room temperature to 210°C for 20 min and at 210°C for 10 min; ETHOSUP, Milestone) and then diluted in 50 ml of water. Ca, P and Ti contents were analysed using an Inductive Coupled Plasma Optical Emission Spectrometer (ICP-OES, iCaP 7000 Series, ThermoScientific; method 990.08; AOAC International, 2006).

To further refine the effects on the pre-caecal digestibility of P when significant, phytate was measured in ileal contents using a commercial assay kit (Megazyme International) and the pre-caecal disappearance of phytate P (PPdisp) determined.

### RNA isolation and RT-PCR assay

Total RNA isolation and real-time RT-PCR assay were performed as described previously in Rousseau *et al*.^(1)^. Concentration and quality of the extracted RNA were assessed by spectrophotometry from 230 to 280 nm, using a Nanodrop 1000 spectrophotometer (Nanodrop Technology). The ratios 260:280 and 260:230 were between 1⋅8 and 2⋅2. The integrity of RNA was assessed by the migration of total RNA on a 1⋅5 % agarose gel. The primers used targeted the following genes (Table 3): P transporters: solute carrier family 20 (phosphate transporter), member 1 (*Gallus gallus* (chicken)) (*SLC20A1*), solute carrier family 34 (Na/phosphate), member 2 (*Gallus gallus* (chicken)) (*SLC34A2)*; Ca transporters: calbindin 1, 28 kDa (*Gallus gallus* (chicken)) (*CALB1*), ATPase, Ca^2+^ transporting, plasma membrane 1 (*Gallus gallus* (chicken)) (*ATP2B1*), solute carrier family 8 (Na/Ca exchanger), member 1 (*Gallus gallus* (chicken)) *(SLC8A1*). The primers used to study the expression of *ATP2B1* and *SLC8A1* have been previously described in Jonchere *et al*.^(15)^. The other primers were specifically designed using Primer BLAST (http://www.ncbi.nlm.nih.gov/tools/primer-blast/). Amplification products were checked by electrophoresis and further sequenced prior to real-time quantitative PCR analysis.

**Table 3.** Primers used for quantitative RT-PCR

Name	Gene symbol	Genbank accession no.	Forward primer sequence (5′→3′)	Reverse primer sequence (5′→3′)	Product size (bp)
Na/phosphate co-transporter 2b	*SLC34A2*	NM_204474	GTCCGTTCACTCTGTTGCCT	TGGGTCCTCTTCTTGCCTTG	242
Na-dependent phosphate transporter 1	*SLC20A1*	XM_003642557	AGGGCAGAAAGGCGTCAA	CGAGGAAGAAGAGAACAGCAGA	104
Calbindin 1, 28 kDa	*CALB1*	NM_205513	CAGGGTGTCAAAATGTGTGC	GCCAGTTCTGCTCGGTAAAG	215
Plasma membrane Ca-transporting ATPase 1	*ATP2B1*	XM_416133	CTGCACTGAAGAAAGCAGATGTTG	GCTGTCATATACGTTTCGTCCCC	130
Na/Ca exchanger 1	*SLC8A1*	NM_001079473	GGATTGTGGAGGTTTGGGAAGG	CTGTTTGCCAGCTCGGTATTTC	137

### Western-blot analysis

Duodenal lysates were prepared as described in Coudert *et al*.^(16)^. Samples were denatured at 75°C for 10 min before loading (80 μg of protein) for migration on 10 % SDS/PAGE gels and then transferred to nitrocellulose membranes. Membranes were blocked in 5 % fat-free milk/PBS, 0⋅1 % Tween for 1 h at room temperature. Membranes were incubated with antibodies against *SLC20A1* (ab177147; Abcam®) 1:5000 for 3 h and against *CALB1* (ab25085; Abcam®) 1:500 overnight. Membranes were then washed several times and incubated for 1 h with goat anti-rabbit secondary antibodies (A21076, AlexaFluor®; Life Technologies). After stripping, membranes were incubated with anti-vinculin antibody (V9131; Sigma) 1:40 000 for 3 h and then incubated with rabbit anti-mouse secondary antibodies (A21065, AlexaFluor®; Life Technologies). Bands were visualised by IR fluorescence using an Odyssey® Imaging System (LI-COR Inc. Biotechnology) and quantified by Odyssey IR imaging system software (Application software, version 1.2).

### Calculations and statistical analyses

The P retained in g/kg body weight (BW) produced was calculated per pen as the sum of P ingested in all phases in g/kg BW produced minus the P excreted in the litter in g/kg BW produced. The P retention efficiency was calculated per pen as the P retained divided by P intake in g/kg BW produced per pen. The AID and PPdisp were determined using the following equation: AID or PPdisp (%) = 100 – (100 × (Ti_Diet_ × *X*_Digesta_)/(Ti_Digesta_ × *X*_Diet_)) where Ti_Diet_ is the Ti concentration of the diet, *X*_Digesta_ the P, Ca or PP concentration of the ileal digesta, Ti_Digesta_ the Ti concentration of the ileal digesta, and *X*_Diet_ the P, Ca or PP concentration of the diet. Determination of the sample size for the study population was based on a power calculation using the estimated mean and standard deviation (35⋅6 (sd 1⋅3) %) for tibia ash content from a previous broiler study^(1)^. Consequently, it was estimated that in order to detect a change in tibia ash content of 4 % at a significance level of 0⋅05, with a power of 90 %, twelve animals per group should be used. A randomised block design with eight blocks was used to test the effect of dietary treatments on growth performance with the pen as the experimental unit for all criteria, except for bone mineralisation where the bird was the experimental unit. Dietary treatment was included in the model as a fixed effect and the block as a random effect. ANOVA were performed on the studied variables for each of the feeding phases using the MIXED procedure of SAS (version 9.2, 2002; SAS Institute Inc.^(^^17^^)^) after the normality of the variables was checked; multiple mean comparisons were done using Tukey's correction. Litter scores were analysed using *χ*^2^ analysis in SAS. Differences were considered significant when *P* < 0⋅05 and *P* < 0⋅10 indicated a statistical trend.

## Results

### Bone mineral status

**Fig. 1.**
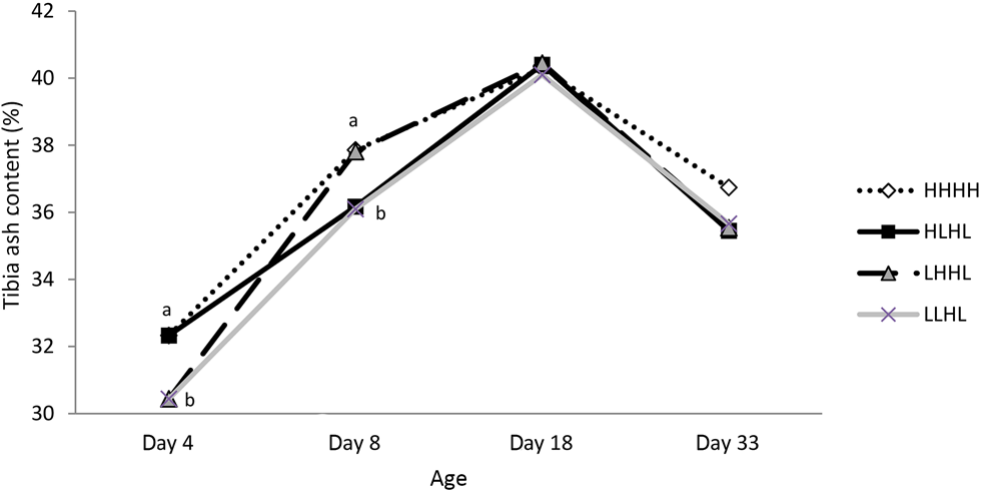
Effect of calcium and non-phytate phosphorus (nPP) diet content on tibia ash content at day 4 (H *v.* L diets from 0 to 4 d), day 8 (HH and LH *v.* HL and LL diets from 4 to 8 d), day 18 (HHH, LHH, HLH and LLH diets from 8 to 18 d) and day 33 (HHHH, LHHL, HLHL and LLHL diets from 18 to 33 d). Values are means (*n* 12). ^a,b^ Least-square mean values within a sampling day with unlike letters were significantly different (*P* < 0⋅05). H, diet high in nPP and calcium; L, diet low in nPP and calcium.

At day 4, tibia ash content decreased with the reduction of P and Ca in the diet (−6 %, *P* *=* 0⋅04; Fig. 1). At day 8, birds receiving the L diet between 5 and 8 d of age (HL and LL treatments) had a lower tibia ash content compared with control birds with the HH treatment (respectively, −4⋅46 and −4⋅65 %, *P* *=* 0⋅005). Birds receiving LH returned to the level of HH at day 8. At day 18 and day 33, no difference in tibia ash content was observed between the experimental groups.

At day 33, the tibia bone mineral density was lower with the LLHL treatment compared with the control treatment (*P* *=* 0⋅045; Table 4). Tibia cortical bone mineral density also tended to be reduced with LLHL treatment compared with HLLL treatment (*P* = 0⋅081). Otherwise, the different depletion−repletion strategies did not affect bone mineral criteria compared with control birds.

**Table 4.** Effect of calcium and non-phytate phosphorus (nPP) on tibia mineralisation at day 33 (Least-square mean values and pooled standard errors)

Dietary treatments*…	HHHH	HLHL	LHHL	LLHL	sem	*P*
*n*	10	10	10	10		
Total bone BMD (g/cm^3^)	1⋅311^a^	1⋅298^a,b^	1⋅304^a,b^	1⋅297^b^	0⋅010	0⋅045
Trabecular bone BMD (g/cm^3^)	1⋅225	1⋅223	1⋅225	1⋅225	0⋅003	0⋅640
Cortical bone BMD (g/cm^3^)	1⋅642	1⋅623	1⋅644	1⋅613	0⋅020	0⋅081

H, high nPP and Ca; L, low nPP and Ca; BMD, bone mineral density.

^a,b^ Least-square mean values within a row with unlike superscript letters were significantly different (*P* < 0⋅05).

* See Table 2.

### Digestibility of phosphorus and calcium and mRNA and protein expression of their transporters

At day 8, the AID of P and Ca as well as PPdisp were higher for birds receiving the L diet between 5 and 8 d of age (HL and LL compared with HH and LH, *P* *≤* 0⋅001, *P* *≤* 0⋅001 and *P* *=* 0⋅002, respectively; Table 5). In parallel, there was a higher mRNA expression of *CALB1* in birds with the LL diet compared with birds receiving HH or LH diets (*P* *≤* 0⋅001). No effect was observed on the protein expression of P transporters or Ca transporters *(P* *>* 0⋅05). At days 18 and 33, diets did not affect P AID or mRNA and protein expression of transporters (Tables 6 and 7). At days 18 and 33, Ca AID was not affected by treatments *(P* *>* 0⋅05).

**Table 5.** Effect of calcium and non-phytate phosphorus (nPP) on the apparent ileal digestibility (AID) of calcium and phosphorus, the ileal disappearance of phytate phosphorus (PPdisp) and normalised relative abundance of transporter mRNA at day 8 (Least-square mean values and pooled standard errors)

Dietary treatments*…	HH	HL	LH	LL	sem	*P*
*n*	8	8	16	16		
Ca AID (%)	22⋅72^b^	46⋅78^a^	22⋅07^b^	49⋅64^a^	1⋅69	<0⋅001
P AID (%)	48⋅34^b^	55⋅68^a^	47⋅16^b^	56⋅57^a^	1⋅08	<0⋅001
PPdisp (%)	23⋅07^b^	31⋅84^a^	19⋅35^b^	33⋅19^a^	2⋅79	0⋅002
mRNA expression						
*n*	12	12	12	12		
*Calb1*	0⋅76^b^	1⋅06^a,b^	0⋅92^b^	1⋅34^a^	0⋅09	<0⋅001
*ATP2B1*	0⋅96	1⋅12	0⋅93	1⋅11	0⋅09	0⋅248
*SLC8A1*	0⋅84	1⋅26	0⋅85	1⋅45	0⋅28	0⋅321
*SLC34A2*	0⋅91	0⋅96	0⋅96	1⋅19	0⋅10	0⋅213
*SLC20A1*	1⋅26	1⋅26	1⋅60	1⋅37	0⋅15	0⋅327
Protein expression						
*n*	6	6	6	6		
Calb1	1⋅31	1⋅32	0⋅70	0⋅79	0⋅86	0⋅453
SLC20A1	2⋅23	0⋅82	1⋅37	0⋅79	2⋅08	0⋅081

H, high nPP and Ca; L, low nPP and Ca.

^a,b^ Least-square mean values within a row with unlike superscript letters were significantly different (*P* < 0⋅05).

* See Table 2.

**Table 6.** Effect of calcium and non-phytate phosphorus (nPP) on the apparent ileal digestibility (AID) of calcium and phosphorus and normalised relative abundance of transporter mRNA at day 18 (Least-square mean values and pooled standard errors)

Dietary treatments*…	HHH	HLH	LHH	LLH	sem	*P*
*n*	8	8	8	16		
Ca AID (%)	35⋅05	34⋅77	34⋅81	37⋅36	4⋅94	0⋅804
P AID (%)	51⋅62	51⋅75	50⋅49	53⋅81	3⋅61	0⋅431
mRNA expression						
*n*	12	12	12	12		
*Calb1*	0⋅95	0⋅91	1⋅00	0⋅99	0⋅29	0⋅440
*ATP2B1*	1⋅17	1⋅03	1⋅06	1⋅17	0⋅35	0⋅657
*SLC8A1*	0⋅25	0⋅23	0⋅15	0⋅16	0⋅15	0⋅559
*SLC34A2*	0⋅94	0⋅84	0⋅97	1⋅01	0⋅23	0⋅392
*SLC20A1*	0⋅29	0⋅32	0⋅19	0⋅24	0⋅18	0⋅346
Protein expression						
*n*	6	6	6	6		
Calb1	0⋅52	0⋅86	0⋅65	0⋅73	0⋅81	0⋅492
SLC20A1	1⋅10	0⋅75	0⋅85	1⋅11	0⋅54	0⋅743

H, high nPP and Ca; L, low nPP and Ca.

* See Table 2.

**Table 7.** Effect of calcium and non-phytate phosphorus (nPP) on the apparent ileal digestibility (AID) of calcium and phosphorus and normalised relative abundance of transporter mRNA and protein at day 33 (Least-square mean values and pooled standard errors)

Dietary treatments*…	HHHH	HLHL	LHHL	LLHL	sem	*P*
*n*	8	8	8	8		
Ca AID (%)	23⋅84	24⋅19	17⋅71	19⋅37	8⋅32	0⋅347
P AID (%)	39⋅27	39⋅74	37⋅11	38⋅54	7⋅62	0⋅918
mRNA expression						
*n*	12	12	12	12		
*Calb1*	1⋅28	1⋅42	1⋅48	1⋅49	0⋅40	0⋅496
*ATP2B1*	0⋅89	1⋅14	1⋅63	1⋅21	0⋅58	0⋅553
*SLC8A1*	0⋅43	0⋅42	0⋅79	0⋅54	0⋅23	0⋅398
*SLC34A2*	0⋅51	0⋅43	0⋅65	0⋅67	0⋅38	0⋅188
*SLC20A1*	0⋅93	0⋅61	0⋅91	0⋅61	0⋅25	0⋅072
Protein expression						
*n*	6	6	6	6		
Calb1	1⋅07	0⋅85	1⋅03	1⋅22	0⋅73	0⋅451
SLC20A1	0⋅70	0⋅92	1⋅46	1⋅35	0⋅82	0⋅373

H, high nPP and Ca; L, low nPP and Ca.

* See Table 2.

### Growth performance

Diets did not affect BW, average daily gain, average daily feed intake or mortality (Table 8). Pododermatitis scores (results not shown) were also not different among treatments. Retention efficiency of P was higher with the depletion–repletion strategies compared with control diets (*P* = 0⋅002), which resulted in lower litter P contents (*P* = 0⋅008; Table 8).

**Table 8.** Effect of calcium and non-phytate phosphorus (nPP) on growth performance of chickens from 0 to 33 d of age and litter quality and phosphorus retention efficiency at day 33 (Least-square mean values and pooled standard errors)

Dietary treatments*…	HHHH	HLHL	LHHL	LLHL	sem	*P*
*n*	8	8	8	8		
ADG (g/d)	64⋅56	63⋅99	65⋅65	65⋅45	1⋅91	0⋅420
ADFI (g/d)	92⋅90	92⋅99	95⋅05	94⋅59	2⋅69	0⋅344
FCR	1⋅461	1⋅471	1⋅465	1⋅456	0⋅015	0⋅261
Mortality (%)	6⋅89	7⋅48	5⋅27	5⋅22	3⋅94	0⋅587
*n*	4	4	4	4		
Litter DM day 33 (%)	48⋅09^a,b^	42⋅49^a^	51⋅41^b^	48⋅01^a,b^	4⋅14	0⋅035
Litter P content (mg/g)	10⋅19^a^	9⋅00^b^	8⋅85^b^	8⋅19^b^	0⋅95	0⋅008
Litter Ca content (mg/g)	13⋅64^a^	11⋅51a^b^	11⋅86a^b^	10⋅75^b^	1⋅27	0⋅001
P retention efficiency (%)	50⋅80^b^	59⋅46^a^	57⋅78^a^	59⋅31^a^	3⋅03	0⋅002

H, high nPP and Ca; L, low nPP and Ca; ADG, average daily gain; ADFI, average daily feed intake; FCR, feed conversion ratio.

^a,b^ Least-square mean values within a row with unlike superscript letters were significantly different (*P* < 0⋅05).

* See Table 2.

## Discussion

### Optimal timing and duration of the first depletion

One of the objectives of the experiment was to study the best timing (from 0 to 4 d of age *v.* from 5 to 8 d of age *v.* from 0 to 8 d of age) and duration (4 d *v.* 8 d) of the initial depletion to induce a permanent adaptation in the birds. The treatments built to answer this question were LHHL, HLHL and LLHL. As expected, a decrease in nPP and Ca content in the diet between 0 and 4 d of age, 5 and 8 d of age or 0 and 8 d of age decreased bone mineral status during the corresponding phase, regardless of the diets received previously. At day 4 and day 8, our results showed a lower tibia ash content in birds with low P and Ca content in the diet, as in Ashwell & Angel^(11)^ for toe ash content. These results agreed with those of Faridi *et al*.^(18)^, who observed by meta-analysis that 4⋅0 g/kg nPP are needed to obtain optimal bone mineralisation until 21 d of age, with 7⋅0 g/kg Ca and 2000 μg/kg of vitamin D_3_.

Birds receiving a diet low in P and Ca increased their P and Ca AID at day 8, but showed lower bone mineralisation than the controls, as observed by Rousseau *et al*.^(1)^. At day 8, this adaptation appeared to be limited to the diet received during the starter phase (day 5−8) regardless of the feed received during the pre-starter phase (days 0−4) with HL and LL leading to similar results. For Ca, this adaptation was linked to an increase of *CALB1* gene expression. Increased AID of Ca linked to increased *CALB1* gene expression^(1)^ was also reported in chicks given a low-Ca diet for a 14-d period. Some authors also observed that a decrease of dietary Ca and nPP levels stimulated the mRNA expression of other Ca transporters: *SLC8A1* and *ATP2B1*, and P transporters: *SLC20A1* and *SLC34A2*^(3,6,19)^. Several facts could explain the discrepancy with our results. For the Ca transporters, *CALB1* has been proven to be the rate-limiting step in Ca absorption through the epithelium, thus *CALB1* expression is highly correlated with intestinal Ca absorption efficiency^(20)^. The lack of effect on protein expression could reflect previous observations that mRNA and protein expression for the same transporter exhibit different temporal patterns^(5)^. Moreover, most experiments find poor correlations between mRNA and protein abundances in the cell due to numerous post-transcriptional regulation mechanisms^(21)^.

As for the lack of effect on P transporter expression, Ca metabolism had been proven to be more closely regulated than P metabolism^(22)^. We hypothesise that Ca was the major mineral deficient in our experiment and drove regulation, thus explaining the lack of effect on P AID at day 18 and day 33 as well as on P transporter protein and mRNA expression. Moreover, P intake through *SLC34A2* was defined as the limiting step of P absorption in the intestine^(9)^. Thus, the regulation of P homeostasis would occur mainly by the stimulation of *SLC34A2* expression. Yan *et al*.^(7)^ and Olukosi *et al*.^(8)^ showed that P content in the diet had to be at 0⋅25 %, lower than used in our L starter diets, in order to enhance the mRNA expression of *SLC34A2*. The regulation of P absorption could also be at the post-translational levels as suggested by Hattenhauer *et al*.^(23)^ in mice, with modifications such as glycosylation, ubiquination or palmitoylation. Alternatively, we could hypothesise that the highest P AID was not linked to a mechanism regulating P transporters, but rather to a mechanism regulating phytic P release through endogenous phytase activity. Phytic P hydrolysis being a major component of the P AID, the pre-caecal disappearance of phytic P was determined in order to better understand this effect. Phytic P disappearance at day 8 was higher for birds fed with the L diet between 5 and 8 d compared with birds fed with the H diet, suggesting an increase in endogenous phytase activity. Onyango *et al*.^(24)^ observed that the activity of mucosal endogenous phytase increased with a reduction of the P content (1⋅2 *v.* 4⋅6 g/kg of nPP) and the supplementation of cholecalciferol (0 *v.* 3320 μg/kg) in the diet.

### Response to a second depletion

It is possible to lower nPP and Ca content in the diet through all phases without affecting growth performance or mortality. Akter *et al*.^(25)^ also observed that Ca and nPP content could be reduced (respectively, 6⋅0 and 3⋅0 g/kg from 0 to 35 d of age) without affecting feed conversion ratio. Delezie *et al*.^(26)^ observed similar results with a reduction of 20−25 % of Ca and nPP dietary contents compared with commercial diets throughout the growth cycle. This result could potentially be explained by the fact that the recommendations are generally set to maximise bone ash^(27)^ which are higher than for growth as reported in a meta-analysis by Faridi *et al*.^(18^^)^.

We hypothesised that birds who had been previously depleted could react better to a second depletion occurring in the finisher phase. A second depletion did not have an impact on bone mineral status. At the end of the finisher phase, no differences in mineral AID were observed between the diets, regardless of the initial period and duration of depletion (see treatments LHHL, HLHL and LLHL). P and Ca depletion in an early phase did not induce a permanent adaptation of P and/or Ca transporter genes, protein expression or growth performance contrary to expectations. It is worth noting that the AID of Ca at day 33 was particularly low. Such low values were previously observed in finisher broilers^(1)^. There is no consensus methodology to assess Ca digestibility and there are frequently inconsistencies through measurements^(28)^. In particular, Ca digestibility is reduced with age in broilers^(29)^. Future studies are needed to understand the reasons for the low digestibility values observed with the direct method used here, among them, the effects of dietary P concentrations and Ca:P ratios on Ca digestibility, the indigestible marker, reverse peristalsis, will be relevant to study. Likewise, Van Krimpen *et al*. (unpublished results) found that birds depleted from 0 to 4 d of age and receiving a control diet from 5 to 37 d of age had the same *SLC34A2*, *CALB1* and *ATP2B1* gene expression as control birds at day 21 and day 37. Conversely, other authors have demonstrated that the implementation of a permanent adaptation was possible. Rousseau *et al*.^(1)^ observed that birds fed with an L diet (0⋅60 % Ca and 0⋅24 % available P) between 10 and 21 d of age and replenished with an H diet (0⋅90 % Ca and 0⋅28 % available P) between 22 and 35 d of age had higher Ca and P AID at day 35 compared with birds fed with diets at recommendations from 10 to 35 d of age. Ashwell & Angel^(11)^ observed that birds depleted during the first 90 h of life had a higher P AID when depleted a second time between day 22 and day 38. These authors used a Ca:nPP ratio of 1⋅64 in the L diet between 0 and 4 d of age, in contrast to 2⋅14 in the present experiment. The Ca and available P contents in the diet between 0 and 4 d of age used by Ashwell & Angel^(11)^ were, respectively, 0⋅59 and 0⋅25 % compared with 0⋅77 and 0⋅36 % in the present experiment. Birds were thus probably more deficient in P than in our experiment. Consequently, it could be hypothesised that P deficiency probably is the inducer of the permanent adaptation.

A decrease in dietary Ca and nPP has been shown to induce regulation in the kidney and bones^(3)^. The expression of Ca and P transporters in the kidney is increased to enhance the Ca and P renal reabsorption and bone resorption is stimulated to release P and Ca^(6)^. A long-term adaptation to the early depletion might have occurred in our experiment in the kidney and bones. Indeed, P retention efficiency was higher for birds that received depletion−repletion treatments while no effect was seen on P AID at day 18 and day 33. The higher P retention efficiency could be linked to higher renal reabsorption of P or increased bone turnover rather than a change in digestibility/absorption.

In the present experiment, we did not observe a long-term adaptation of the animals to an early depletion, while P retention efficiency was around 51 % in the control diet. Van Krimpen *et al*. (unpublished results) also did not observe a long-term adaptation with similar P retention efficiency values. In contrast, Ashwell & Angel^(11)^ observed that birds depleted during the first 90 h of life exhibited higher P absorption at 38 d compared with control birds when both groups were fed with a low finisher diet. The P retention efficiency in this study was 35⋅3 % in the control diet, compared with 47⋅1 % in the low pre-starter diet accompanied by a higher total P intake for the control birds (11⋅9 g/kg BW). As suggested by Van Krimpen *et al*. (unpublished results) the apparent long-term adaptation observed by Ashwell & Angel^(11)^ could result from a reduced P retention efficiency in the control diet rather than an enhancement resulting from the depletion during the pre-starter phase.

### Necessity of a repletion

One of our preliminary hypotheses was that a repletion phase was necessary in order to catch up in bone mineralisation. Birds depleted from 0 to 4 d of age had the same bone mineral status at day 8 compared with control birds when fed with the H diet between 5 and 8 d of age. Likewise, birds depleted from 0 to 8 d of age or 5 to 8 d of age showed similar bone criteria at day 18 compared with control diets, when replenished between 9 and 18 d of age. The birds seemed to be able to catch up in bone mineralisation after 4 or 8 d on a control diet. Whatever the type of initial depletion, repletion with the H diet led to similar P and Ca AID at 18 d of age. As a result, higher Ca and nPP content in the diet was the explanation of a catch up in bone mineralisation rather than a permanent adaptation of birds to increase P and Ca digestive utilisation.

In the finisher phase, the birds were able to maintain a mineral status at the control levels when fed the L diet. It had been demonstrated that P and Ca requirements for bone growth and metabolism decrease with age^(30)^ and the dietary Ca and nPP content were reduced accordingly in our experiment. Moreover, the decrease in nPP and Ca dietary content was less severe for the birds during the finisher phases 1 and 2 (respectively, −21 and −25 % compared with −30 % during the pre-starter and starter phase). Interestingly, the most depleted programme, LLHL, led to lower tibia bone mineral density without effects on tibia ash content. Bone mineral content discriminated between the dietary treatments with more sensitivity than classical bone measures as seen in Angel *et al*.^(31)^ and Bradbury *et al*.^(22)^. Interestingly, cortical bone mineral density followed the same trend to discriminate between the treatments compared with total bone mineral density. However, trabecular bone mineral density was not affected by dietary treatment. Likewise, Kim *et al*.^(32)^ and Jendral *et al*.^(33)^ found that total and cortical bone mineral density discriminated between the treatments with no effect on trabecular bone mineral density in, respectively, broiler chicks at day 21 and hens at 65 weeks of age. As stated by these authors, these results could be due to excessive bone resorption from the endosteal surface located at the inner edge of the cortical shell. The analysed feed composition showed that the Ca:nPP ratio in the L diet during the finisher phase was 2⋅65 compared with 2⋅20 as formulated. As a result, the Ca intake compared with the nPP intake was much higher than formulated during this phase. The higher ratio could reduce P availability and then increase bone resorption.

### Conclusion

Reducing P and Ca content in the diet did not have an impact on growth performance. Despite reduced bone mineralisation during the initial depletion phase, the birds were able to catch up in bone mineralisation at day 8 or at day 18, after being fed a diet at commercial Ca and nPP levels during, respectively, the starter or grower phase. It is then possible to lower nPP and Ca in the diet during the finisher period, without affecting growth performance or bone mineralisation. Our results demonstrated that it was not necessary to replenish the diets during the finishing period in order to ensure acceptable bone mineralisation, thus limiting the environmental impact of the production. The birds adapted to a diet low in P and Ca content by increasing their P and Ca AID accompanied by stimulation of the mRNA expression of the Ca transporter, *CALB1*. This adaptation appeared to be limited to the starter phase for birds receiving the depleted diet. None of our initial depletion strategies led to a long-lasting adaptation. All of the depletion–repletion strategies used improved litter quality. However, the LLHL programme showed the most promising results, with optimal growth performance and the largest decrease in environmental and economic impacts.
